# Novel evaluation of upper-limb motor performance after stroke based on normal reaching movement model

**DOI:** 10.1186/s12984-023-01189-6

**Published:** 2023-05-25

**Authors:** James Hyungsup Moon, Jongbum Kim, Yeji Hwang, Sungho Jang, Jonghyun Kim

**Affiliations:** 1 School of Mechanical Engineering, Sungkyunkwan University, Suwon-Si, Gyeonggi-Do 16419 Republic of Korea; 2 Department of Robotics and Mechatronics Engineering, Daegu Gyeongbuk Institute of Science and Technology (DGIST), Daegu, 42988 Republic of Korea; 3 Department of Physical Medicine and Rehabilitation, College of Medicine, Yeungnam University, Daegu, 42415 Republic of Korea

**Keywords:** Reaching movement, Rehabilitation robot, Motor performance evaluation, Human model

## Abstract

**Background:**

Upper-limb rehabilitation robots provide repetitive reaching movement training to post-stroke patients. Beyond a pre-determined set of movements, a robot-aided training protocol requires optimization to account for the individuals’ unique motor characteristics. Therefore, an objective evaluation method should consider the pre-stroke motor performance of the affected arm to compare one’s performance relative to normalcy**.** However, no study has attempted to evaluate performance based on an individual’s normal performance. Herein, we present a novel method for evaluating upper limb motor performance after a stroke based on a normal reaching movement model.

**Methods:**

To represent the normal reaching performance of individuals, we opted for three candidate models: (1) Fitts’ law for the speed-accuracy relationship, (2) the Almanji model for the mouse-pointing task of cerebral palsy, and (3) our proposed model. We first obtained the kinematic data of healthy (*n* = 12) and post-stroke (*n* = 7) subjects with a robot to validate the model and evaluation method and conducted a pilot study with a group of post-stroke patients (*n* = 12) in a clinical setting. Using the models obtained from the reaching performance of the less-affected arm, we predicted the patients’ normal reaching performance to set the standard for evaluating the affected arm.

**Results:**

We verified that the proposed normal reaching model identifies the reaching of all healthy (*n* = 12) and less-affected arm (*n* = 19; 16 of them showed an *R*^2^ > 0.7) but did not identify erroneous reaching of the affected arm. Furthermore, our evaluation method intuitively and visually demonstrated the unique motor characteristics of the affected arms.

**Conclusions:**

The proposed method can be used to evaluate an individual’s reaching characteristics based on an individuals normal reaching model. It has the potential to provide individualized training by prioritizing a set of reaching movements.

## Background

Reaching movement (RM), among the most important recovery goals of stroke rehabilitation, represents inter-joint coordination in activities of daily living [1]. Rehabilitation robots can provide repetitive reaching training to post-stroke patients, whereas conventional rehabilitation modalities are labor intensive [2–4]. Beyond a predetermined and fixed set of movements, the training protocol will be more effective when optimized based on patient-specific needs for motor recovery [5–7]. Hence, the individualization of robotic therapy is required to account for an individual’s unique motor characteristics, resulting in varying motor performance and learning capacities [8, 9]. Therefore, an evaluation method that can objectively identify a patient’s motor characteristics is required for individualized robot-aided reaching training.

A well-established rehabilitation process entails the evaluation of the progress and effect of robotic intervention on an individual’s rehabilitation goals [10, 11]. Because the process would be directing an impaired task to progress toward a normal task [12–14], the evaluation of the RM needs to address the level of motor impairment of the affected limb based on the individual’s pre-stroke motor capacity, to demonstrate one’s reaching performance level relative to normalcy. Particularly, it is well known that understanding one’s desired recovery is a significant biomarker for prescribing the rehabilitation dose [15]. Hence, normal reaching characteristic information, which varies across individuals [8], needs to be the standard that sets a basis for the patients’ current reaching performance.

Several studies have utilized evaluation methods based on kinematic characteristics for the upper-limb motor ability and function in post-stroke patients during robot-aided therapy [9, 16–20] and have characterized the relative motor deficits of the affected limb in various directions or distances. However, they did not evaluate motor characteristics based on an individual’s normal performance; thus, the training effect was relatively evaluated before and after treatment, which does not consider the individual’s rehabilitation goal [9, 16–20]. In contrast, evaluating a patient’s current performance based on their normal performance provides an individually scaled motor deficit, which results in an objective evaluation of the robot-aided therapeutic effect. Therefore, it is necessary to understand the normal (original) reaching performance before stroke onset in a patient-specific manner to enhance the evaluations of individualized robotic reaching training.

However, it is unfeasible for patients to possess knowledge or data on their quantified normal reaching performance. Thus, an estimation method that can identify the before-onset reaching abilities of individuals is required. A plausible approach for estimating the patients’ normal reaching performance is to model the performance based on their less-affected arm; however, to the best of our knowledge, no such attempt has been made to date. Based on several studies that reported the similarity in motor performance between the post-stroke patients’ less-affected arms and healthy individuals’ arms [21–23], we posit that this approach could represent the normal reaching ability of post-stroke individuals. Here, the model needs to (1) represent one’s normal reaching ability well, and (2) not characterize one’s erroneous RM. Such a model can take task information (i.e., target distance) and human kinematic information (i.e., movement speed) as inputs and predict the movement time of the normal RM as an output.

Despite the above approach, the issue of how to model the less-affected arm persists. Several statistical models have captured quantifiable reaching characteristics [24–26]; however, to the best of our knowledge, there is no appropriate model for evaluating the normal RM of stroke. Fitts’ law, which is the most well-known reaching model [24], has been applied to quantify the reaching performance of stroke survivors. Although this method was simple and feasible for motor recovery, the results showed model inaccuracy under Fitts’ law [27]. This poor accuracy of Fitts’ law was also indicated by another study that modeled the reaching-related movements of patients with cerebral palsy (CP) [26]. To improve accuracy, a novel model that showed good accuracy was developed for CP movements [26]. However, the model was too complex and was not validated by the RM of stroke survivors.

This study developed a novel evaluation method for individualized reaching training. We first identified a novel normal reaching model and formulated a method for evaluating motor deficits based on the proposed model of the less-affected arm. We then utilized the evaluated metrics to express an individual’s motor deficit as a contour map of the workspace [19, 20]. To validate the results, statistical analyses were conducted using experimental RM data collected from 12 healthy subjects and 19 patients with stroke. The results showed that the proposed method could enable individually scaled RM evaluation by comparing the performance of the affected arm with that of the normal reaching model of the less-affected arm.

## Methods

### Individually scaled evaluation method

**Fig. 1:**
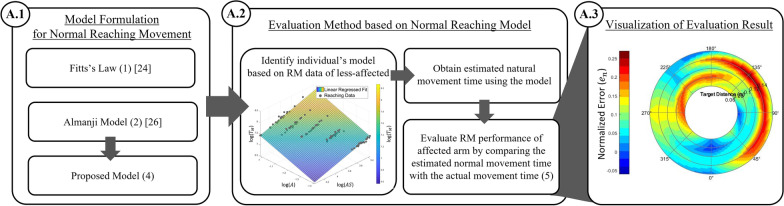
Development process of individually scaled evaluation method. The blocks of the development procedures are denoted with marked circles in the following sections in Methods. Once an appropriate model for a normal RM is established, A.2 and A.3 are the general steps for utilizing the proposed evaluation method

In this subsection, we present candidate reaching models for describing the normal RM and explain how we derived the normal reaching model for post-stroke patients. Subsequently, based on the model, we defined the individually scaled performance index and visually mapped it for the evaluation method (Fig. 1).

#### Model formulation for normal reaching movement

Reaching models quantitatively describe an individual’s unique performance, and such models use kinematic variables as the inputs and yield the movement time as the outputs. To establish an appropriate normal reaching model, we first studied the characteristics of two existing models: Fitts’ law and a reaching-related model of CP [19–21]. We then deduced a normal reaching model using these models.

Fitts’ law, the best-known reaching model [24], is described as follows:

1 $${T}_{M}=a+b\times {\mathrm{log}}_{2}\left(\frac{2A}{W}\right)$$

where *T*_*M*_ denotes the movement time, *A* and *W* are the reaching target distance and width, respectively, and *a* and *b* are the intercept and slope of the linear model, respectively. Fitts’ law was not originally proposed to accurately capture the characteristics of RM, but instead focused on the speed–accuracy tradeoff in RM with various target sizes and lengths [24]. Hence, (1) contains only target-related environmental variables and does not include any behavioral variables (those related to an individual’s behavior) that could also affect *T*_*M*_. For instance, there is no variable in (1) that reflects the fluctuation of the movement speed with the same target (*A* and *W*). Notably, the target size is usually fixed in a robotic reaching training task for rehabilitation because the fixed target size is recommended by Fitts’ law [28], and the speed–accuracy tradeoff is not the main issue [4, 20, 28, 29]. This implies that (1) is insufficient to accurately model RM characteristics, and this was supported by an attempt to apply (1) to describe RM in stroke patients; the result showed poor modeling accuracy [27]. It should be noted that there is another form of Fitts’ law, including the error rate [30]. However, we did not use the form with the error rate to consider their impaired movement [20, 29, 31] because the robotic reaching tasks for post-stroke patients have been a type of errorless form [30] of reaching, such as disc or pin transfer tasks [24, 32].

To improve and maximize the reaching modeling accuracy, one study reported a sophisticated reaching model that incorporated both environmental and behavioral variables, even when considering erroneous human behaviors [26]. We refer to this model as the Almanji model, and it accounts for a reaching-related (mouse pointing) movement in CP and is expressed as follows [26]:

2 $${T}_{M}={e}^{k}\times {A}^{\alpha }\times {AS}^{\beta }\times {\left(EC+1\right)}^{c}{\times {\left(NS+1\right)}^{d}\times (NSO+1)}^{e}\times {CI}^{f}$$

where *AS* denotes the average speed of movement*; EC* the erroneous clicks; *NS* the number of submovements [26]; *NSO* the number of slip-offs; *CI* the curvature index [26], and $\alpha , \beta$, *c*, *d*, *e* and $k$ are the model parameters. Model (2) is more appropriate than (1) in terms of the modeling accuracy. However, it still has limitations in the target movement and objective of the model. Regarding the former, the target movement of (2) was a mouse-pointing task; thus, (2) contained variables related to mouse clicks, including *EC* and *NSO*. However, our target reaching task does not require a mouse click to discriminate the success of the task, hence *EC* and *NSO* were not of interest. Hence, model (2) must be simplified as follows:

3 $${T}_{M}={e}^{k}\times {A}^{\alpha }\times {AS}^{\beta }\times {(NS+1)}^{d}{\times CI}^{f}$$

Despite its simplicity, model (3) remains unsuitable because of the model objective of its original model (2); it does not aim to describe normal reaching, but rather RM with erroneous human behaviors. It is well known that the normal RM (when one reaches the target without any erroneous behaviors) is ideally a straight movement with minimal submovement [26], and *CI* and *NS* in (2) can be considered constants (1 for *CI* and 3 for *NS* [33]). Therefore, the simplified model (3) was further modified to derive the normal reaching model as follows:

4 $${T}_{M}={\widehat{e}}^{k}\times {A}^{\alpha }\times {AS}^{\beta }$$

Equation (4) implies that *T*_*M*_ of the normal reaching is determined by reaching the target distance (*A*) and average moving speed (*AS*). In contrast to Fitts’ law (1), the proposed model (4) includes *AS*. The effect of the moving speed variation during RM is considered to improve the modeling accuracy of normal RM. Notably, (4) does not include the target width (*W*) because of the fixed target size in the robotic reaching training task.

#### Evaluation method based on normal reaching model

**Fig. 2:**
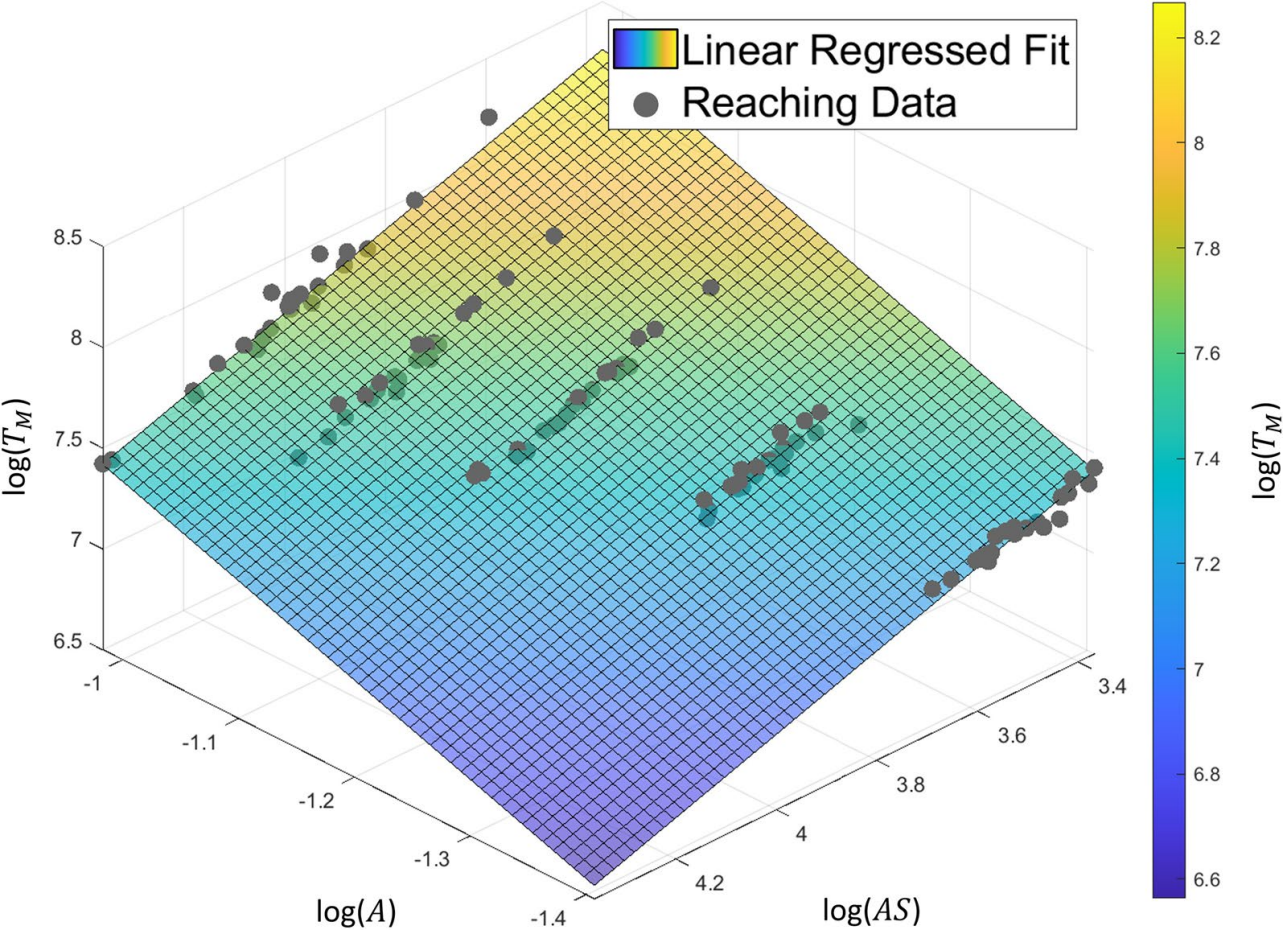
Example linear regression fit for a reaching model. Reaching parameters such as the movement time ($${T}_{m})$$, target distance (*A*) and average speed (AS) were logarithmically transformed. The colored plane represents the plane of the model parameters that fit the acquired data (gray dots)

Using the model above, we developed a novel individually scaled evaluation method for post-stroke patients; the method involves the following steps: (1) establishing an individual’s normal reaching model based on (4) of one’s less-affected arm (Fig. 2), (2) obtaining the estimated ideal movement time for normal reaching (*T*_*M,e*_) using the model, and (3) evaluating the reaching performance of the affected arm by comparing *T*_*M,e*_ with the actual movement time (*T*_*M,a*_). When *T*_*M,a*_ significantly deviates from *T*_*M,e*_, the discrepancy implies that RM is abnormal because of the presence of erroneous behavior such as anomalous *CI* or *NS*, violating the definition of (4).

To identify an individual-specific normal reaching model using (4), we used several RM datasets of the less-affected arm in various directions and distances and conducted a multiple linear regression analysis. Here, we defined the arm ipsilateral to the lesion as the less-affected arm and the contralateral arm as the affected arm in hemiplegia. Although the less-affected arms of stroke patients are not completely free from impairment, they are expected to behave closer to the condition prior to stroke onset than the affected arm. One study reported that the movement time distributions of healthy participants’ arms and post-stroke participants’ less-affected arms were similar [21], and another study reported no significant differences in the movement time between healthy and post-stroke less-affected arms [22, 23]. Hence, the RM data of the less-affected arm could be a reliable source for predicting an individual’s normal reaching unless one suffers from diplegic symptoms.

Using an established normal reaching model, we set an individually scaled standard for normal RM by estimating the ideal movement time (*T*_*M,e*_) for each condition. Subsequently, the actual movement time (*T*_*M,a*_) of the affected arm at the corresponding movement condition was compared to *T*_*M,e*_ by defining a performance index normalized for error as follows:

5 $${e}_{n}(i)= \frac{{T}_{M, a}(i) -{T}_{M,e}(i)}{{T}_{M,e}(i)}$$

where *i* denotes the index of the target. As the denominator term *T*_*M,e*_ normalizes the discrepancy, the normalized error ${e}_{n}$ (5) provides an individually scaled quantity that reflects the abnormality level. As the stroke reaching procedure is affected by sensorimotor noise [34, 35], the *T*_*M,a*_ of the stroke would increase and exhibit more discrepancies from *T*_*M,e*_.

#### Visualization of evaluation results

**Fig. 3:**
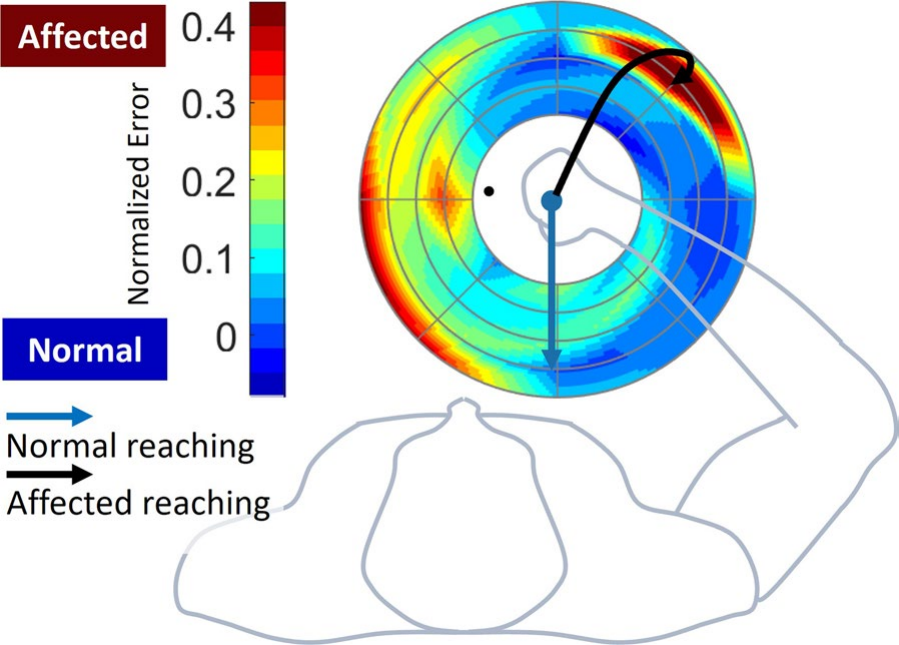
Example assessment profile mapping. The color gradient represents the normalized error level defined by the proposed evaluation method. The blue and black arrows are the example trajectories of the normal and affected reaching, respectively, whereas the red and blue shades in the contour represent the affected and normal reaching performance on the reaching targets respectively

We constructed an evaluation map (Fig. 3) based on the acquired index ${e}_{n}$ (5) throughout the workspace. A high deviation of the reaching trajectory, which is represented by a black arrow line, results in a high ${e}_{n}$, which is represented in the red-colored region according to the spectrum range.

The proposed visualization method allows the evaluation of an individual’s motor characteristics and prescription of performance-based training [4, 19, 36]; thus, we may implement adaptive scheduling to prioritize reaching training and prevent overtraining of a satisfactory reaching condition, where the “labor in vain” problem could arise with random scheduling [36]. This visualization is particularly beneficial in multi-distance-directional reaching training environments. Solely mapping the reaching performance with the movement time can be problematic because a short movement time is generally preferred. Because further targets are likely to have long movement times, the training could be biased toward outer-most targets regardless of the actual reaching performance in the workspace. In contrast, the proposed visualization using the normalized index (5) can objectively portray the reaching performance globally in a spatial sense.

### Experiments

#### Experimental design

We conducted two experiments to validate the proposed normal reaching model and verify the feasibility of the developed evaluation method. For the former, we compared candidate reaching models including the Fitts (1), Almanji (3), and proposed (4) models by examining the fit degree of the RM data of the healthy subjects. This is because their reaching tasks could be considered normal. It should be noted that we used simplified (3) instead of (2), because our reaching task required fewer conditions than the original mouse-pointing task. For the latter, post-stroke subjects performed the reaching task with the less-affected and affected arms. We obtained each patient’s normal reaching model (4) based on the RM data of the less-affected arm and evaluated the performance of the affected arm using the evaluation map with normalized error (5). Additionally, as a pilot study, we conducted the same procedures as the latter experiment with different post-stroke participants in an actual clinical setting to further justify the proposed method in an actual clinical setting with a condensed procedure. Here, the participants performed fewer number of RM trials, which was sufficient to form reaching models and evaluate the affected reaching.

**Fig. 4:**
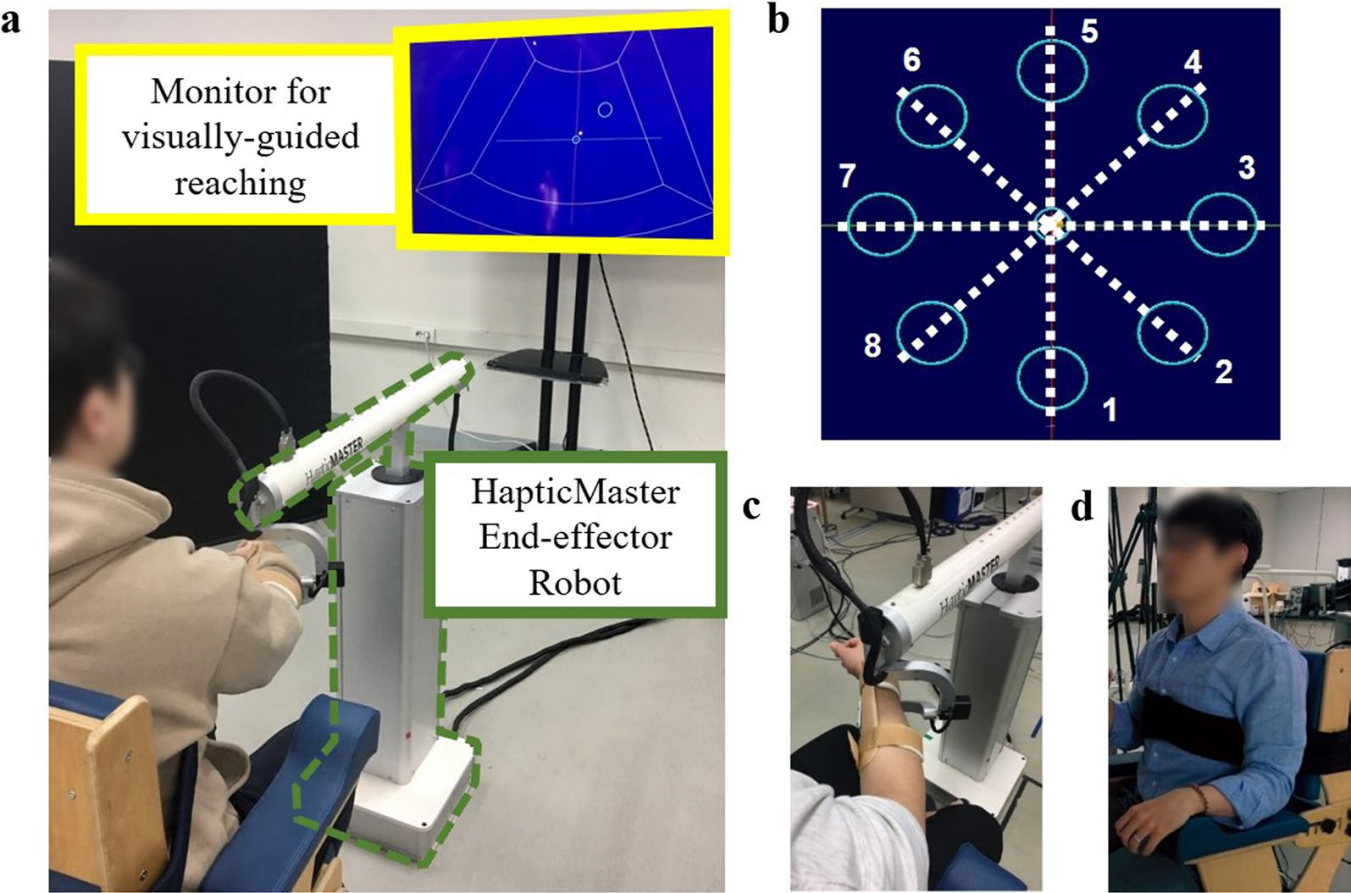
Experimental setup for validation experiments. **a** Visually guided reaching system with an end-effector–type robot. **b** Sample reaching targets in eight directions. **c** Gimbal with gravity-compensated forearm rest. **d** Trunk-constraining chair

Figure 4 shows the experimental setup used for the validation experiments. All subjects sat on a trunk-constraining chair and performed a visually guided reaching task with HapticMaster (MOOG, Netherlands), a three-degrees-of-freedom (DOF) end-effector–type robot (Fig. 4a). The robot recorded the movement time, position, and velocity of its end-effector for each RM trial at a 75-Hz sampling rate. Because the subjects rested their forearm on the gimbal of the robot, supporting their arm against gravity (Fig. 4c), the reaching task was constrained to motion with 2DOF. The robot was located 30 cm between the gimbal and subject (Fig. 4a). The chair had a strap to constrain the trunk movement and prevent compensatory movements (Fig. 4d). For the reaching task, a monitor was set 3 m away from the seat (Fig. 4a), and the subjects performed start-to-target reaching according to custom-made visual guidance software developed in Visual Studio (Microsoft, USA) (Fig. 4b).

**Fig. 5:**
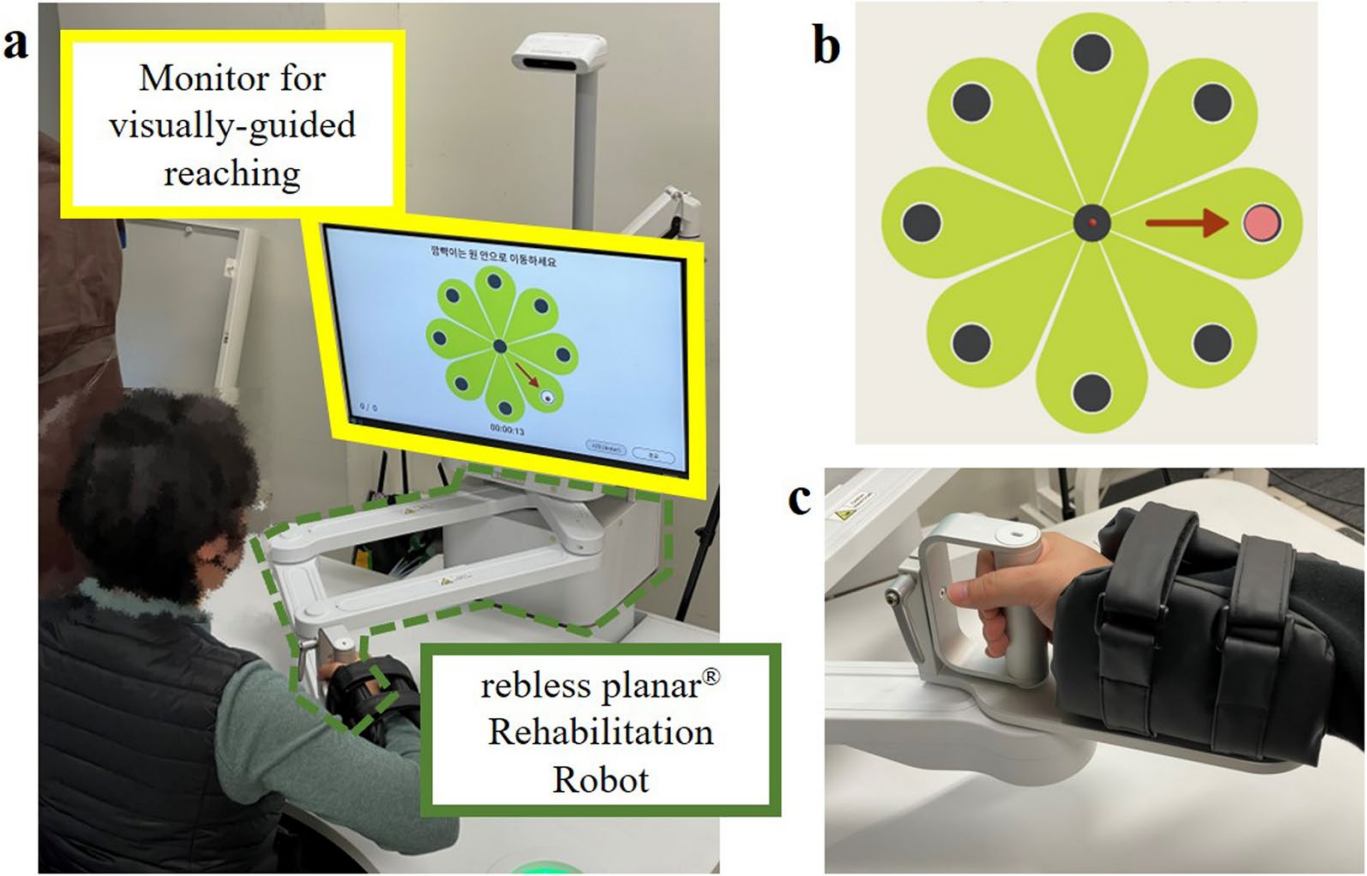
Experimental setup for the pilot study. **a** End-effector type upper-limb rehabilitation robot. **b** Sample reaching targets in eight directions. **c** Forearm rest of the rehabilitation robot

Figure 5 shows the experimental setup of the pilot study. All subjects sat on a regular chair and performed a visually guided reaching task with rebless planar® (H-Robotics, South Korea), a 2-DOF end-effector–type upper limb rehabilitation robot (Fig. 5a). The robot recorded the movement time, position, and velocity of its end-effector for each RM trial at a 30-Hz sampling rate. The subjects rested their forearm on the robot end-effector to support their arm against gravity (Fig. 5c) and performed start-to-target reaching according to the custom-made visual guidance software developed in Unity (Unity Technologies, USA) (Fig. 5b).

#### Participants

For the validation experiments, twelve healthy (26.2 ± 2.7 years; two females, 10 males) and seven stroke survivors (58.4 ± 8.0 years; two females, five males) participated in this study (Table 1). All survivors had an affected arm on their right side. The inclusion criteria for stroke subjects were as follows: (1) hemiplegia; (2) unilateral stroke; (3) no signs of visual, spatial, or sensory deficits; and (4) mini-mental status examination scores greater than 24, indicating that the subjects could understand the instructions for the experiments. The exclusion criteria were as follows: (1) habitual shoulder dislocation; (2) musculoskeletal disorders; and (3) Parkinson’s disease, aphasia, apraxia, diabetes. This study was approved by the local institutional review board (DGIST-150408-HR-008- 02).

**Table 1:** Participants details of affected arm in validation experiments

Subjects	Sex	Age	Time since stroke [months]	Lesion type	MAS				MMT				MBC
					Shoulder	Elbow	Wrist	Finger	Shoulder	Elbow	Wrist	Finger	
S1	M	57	88	H	0	0	0	0	4 +	4 +	4 +	4 +	6
S2	F	58	86	H	1 +	1 +	1 +	1 +	3 +	3 +	2-	2 −	4
S3	M	63	38	H	2 +	2 +	2 +	2 +	3 +	3 +	3 +	3 +	5
S4	M	39	110	I	2	2	2	2	2 +	3	0	0	1
S5	M	58	113	H	1	1	1	1	2 +	2	0	0	1
S6	F	59	41	H	1 −	1 −	1 −	1 −	3 +	3 +	3 −	3 +	4
S7	M	61	19	I	0	0	0	0	4 −	3 +	4	4	6

MAS denotes modified Ashworth scale; MMT manual muscle test; MBC modified Brunnstrom classification; and H and I are hemorrhagic and ischemic stroke, respectively

Twelve stroke survivors (67.5 ± 6.8 years; four females, eight males) participated in this pilot study (Table 2). Six stroke survivors had an affected arm on their right side. The inclusion criteria for the stroke subjects were as follows: (1) hemiplegia, (2) upper limb modified Ashworth scale score < 3, and and (3) post-stroke age > 19 years. The exclusion criteria were as follows: (1) cognitive deficits or aphasia, (2) internal medical conditions, (3) neurological or musculoskeletal disorders, and (4) other conditions that inhibit upper-limb rehabilitation exercises. This study was approved by the institutional review board of Asan Medical Center (IRB No. 2022-0981).

**Table 2:** Participant details of affected arm in pilot study

Subjects	Sex	Age	Time since stroke [months]	Lesion type	Hemi-side	MAS				FMA-UE
						Elbow flexor	Elbow extensor	Wrist flexor	Wrist extensor	
P1	M	69	52	I	L	1.5	0	1	0	42
P2	F	65	81	H	R	1	0	0	0	39
P3	M	64	72	H	L	1	0	1	0	39
P4	F	61	96	I	R	0	0	0	0	41
P5	M	67	78	I	R	0	0	0	0	43
P6	M	81	124	I	R	1	0	0	1	40
P7	M	54	61	H	R	2	0	0	0	33
P8	M	74	111	I	L	1.5	0	1.5	0	33
P9	M	70	104	I	L	2	0	1.5	1	38
P10	F	67	121	H	R	1	0	1.5	0	25
P11	F	63	75	H	L	0	0	0	0	35
P12	M	75	113	I	L	0	0	0	0	34

FMA-UE denotes Fugl-Meyer upper extremity assessment; MAS modified Ashworth scale; H and I hemorrhagic and ischemic strokes, respectively; and L and R denote the left and right sides, respectively

It is noteworthy that the stroke survivor group was older than the healthy group while there were no inclusion/exclusion criteria for this age difference. Older adults generally have longer movement times [37, 38], more variable velocities [39] and more corrective movements [40] than young subjects.

#### Protocols

In the validation experiments, the subjects sat on a trunk-constraining chair to which a strap was attached (Fig. 4d). The subject’s arm was secured using a gimbal (Fig. 4c). On the monitor, a cursor representing the position of the robot was displayed at the center, which was the initial position for the center-out reaching task (Fig. 4b). For each visually guided reaching task, the subjects were asked to move the cursor on the monitor to reach the target at their preferred speed (Fig. 4b). The reaching task consisted of three blocks of 40 different movement conditions (eight directions, five target distances), resulting in 120 trials (Table 3). For each block, the target randomly appeared once at every possible location. Once the cursor remained in the target position for 1 s, the trial was considered successful, and the robot returned the subject’s arm to the initial position. The reaching completion time was not constrained, and pauses or corrective movements were allowed. However, certain reaching trials were skipped if the subjects could not reach the target. The subjects were instructed to restrain their trunk movements during the reaching task and were given a rest period between blocks. The healthy subjects performed the reaching task with their dominant arm, whereas the stroke subjects performed the task with both the less-affected and affected arms. For the pilot study, the protocols were different in the chair setting and number of reaching trials. The subjects sat on a regular chair (Fig. 5a); instead, they were asked to constrain their trunk movements as much as possible. When conducting the pilot study, we recognized that the model formulation did not require all 120 trials of the RM data. Thus, in the pilot study, the reaching task consisted of blocks of 24 different movement conditions (eight directions, three target distances), resulting in 24 trials for the less-affected arm and 48 trials for the affected arm (Table 3).

**Table 3:** Summary of experiment configuration

Experiments	Validation experiments		Pilot study
Participants	Healthy (*n* = 12)	Post-stroke (*n* = 7)	Post-stroke (*n* = 12)
Target directions	Eight directions (0°, 45°, 90°, 135°, 180°, 225°, 270°, and 315°)		
Target distances	Five distances (6, 8, 10, 12, and 14 cm) with fixed target width (2 cm)		Three distances (6, 10, and 14 cm) with fixed target width (3 cm)
Number of reaching trials	120 trials with the dominant arm	120 trials with less-affected arm 120 trials with affected arm	24 trials with less-affected arm 48 trials with the affected arm

#### Data analysis

During the reaching task, we collected the movement time, position, and velocity and post-processed the collected data to obtain other kinematic variables, such *AS*, *CI,* and *NS*. It should be noted that we calculated *NS* using the optimal submovement decomposition method to avoid exaggeration [33].

In the normal reaching modeling process, we rejected the RM data with outlier *CI* beyond two interquartile ranges (Q1–Q3). Notably, the number of reaching trials used and the number of the outliers were 115.0 ± 3.1, 5.0 ± 3.1 for the healthy participants, 107.8 ± 13.2, 7.3 ± 3.5 for the post-stroke participants in the validation experiment, and 23.4 ± 0.8, 0.6 ± 0.8 for the pilot study, respectively. It is well-known that *CI* is a dominant contributor to human effects on goal-directed movements [26]. We considered that such an extremely high *CI* represented reaching by mistake, which would not be normal reaching.

To evaluate RM using (5) based on the discrepancy between normal and erroneous RM, an appropriate normal reaching model must have the model characteristics: the model should identify normal RM, but it should not accurately identify reaching with erroneous behaviors. Hence, we analyzed the RM data to validate the proposed model (4) using the following steps. First, we evaluated the candidate reaching models using healthy RM data to determine whether the models could predict the healthy movement time, which is identical to the movement time of normal reaching. For this, we adopted the Akaike information criterion (AIC) and coefficient of determination (*R*^*2*^) for each model. Along with *R*^*2*^, which provides a general idea of how well a model fits the data (larger indicates a better fit), AIC, which measures the predictive accuracy of the model (smaller indicates higher accuracy) [41, 42], has been used for model selection studies involving human movement [26, 43]. Using the same indices (AIC and *R*^*2*^), we validated the candidate models using the RM data of the less-affected and affected arms of the stroke patients to determine whether the patients’ data could be used to formulate a normal reaching model.

Moreover, using the affected RM data, we further inspected model fitting to determine whether the candidate models identified erroneous reaching because we posited that an appropriate normal reaching model would not predict erroneous reaching movement times. We first constructed reaching models with the affected RM data to predict the movement time of the affected reaching (*T*_*M,aff*_) and observed the relationship between the residuals (*T*_*M,a*_ − *T*_*M,aff*_) and error-related parameters (*NS* and *CI*). It should be noted that varying *NS* and larger *CI* values were associated with erroneous reaching. We then calculated the mean magnitude of the residuals for each model to compare the model fitting in the erroneous reaching, where a low magnitude indicates an accurate model fitting in the affected reaching, which is not preferred for the normal reaching model.

The model parameters were estimated using linear regression with logarithmic transformation, in which the movement time was the independent variable. All regression analyses, including *R*^*2*^ and statistical tests for AIC (non-parametric tests: Friedman test and Wilcoxon signed-rank test), were conducted using IBM SPSS (IBM Corporation, USA). AIC was calculated using the *fitlm* function in MATLAB R2020b (MathWorks, USA).

After validating the proposed normal reaching model, we visualized the reaching evaluation results for stroke based on ${e}_{n}$ (5) as well as ${T}_{m,a}$ (movement time) using MATLAB in both the validation experiments and pilot study. For the former visualization with ${e}_{n}$, the spectrum range of the map (blue to red) was set to the minimum ${e}_{n}$ and 95th percentile value was set as the maximum. This clipped maximum prevents an extremely high ${e}_{n}$ from an overly inflated axis range, which does not capture the overall reaching characteristic. We used the mean of ${e}_{n}\left(i\right)$ obtained by repeated reaching tasks to ensure better reliability. Additionally, we visualized the contours based on the ${T}_{m,a}$ with the same data used for the corresponding ${e}_{n}$ maps to show the distinctiveness of the proposed index ${e}_{n}$.

## Results

### Normal reaching model performance

#### Predictive accuracy

**Fig. 6:**
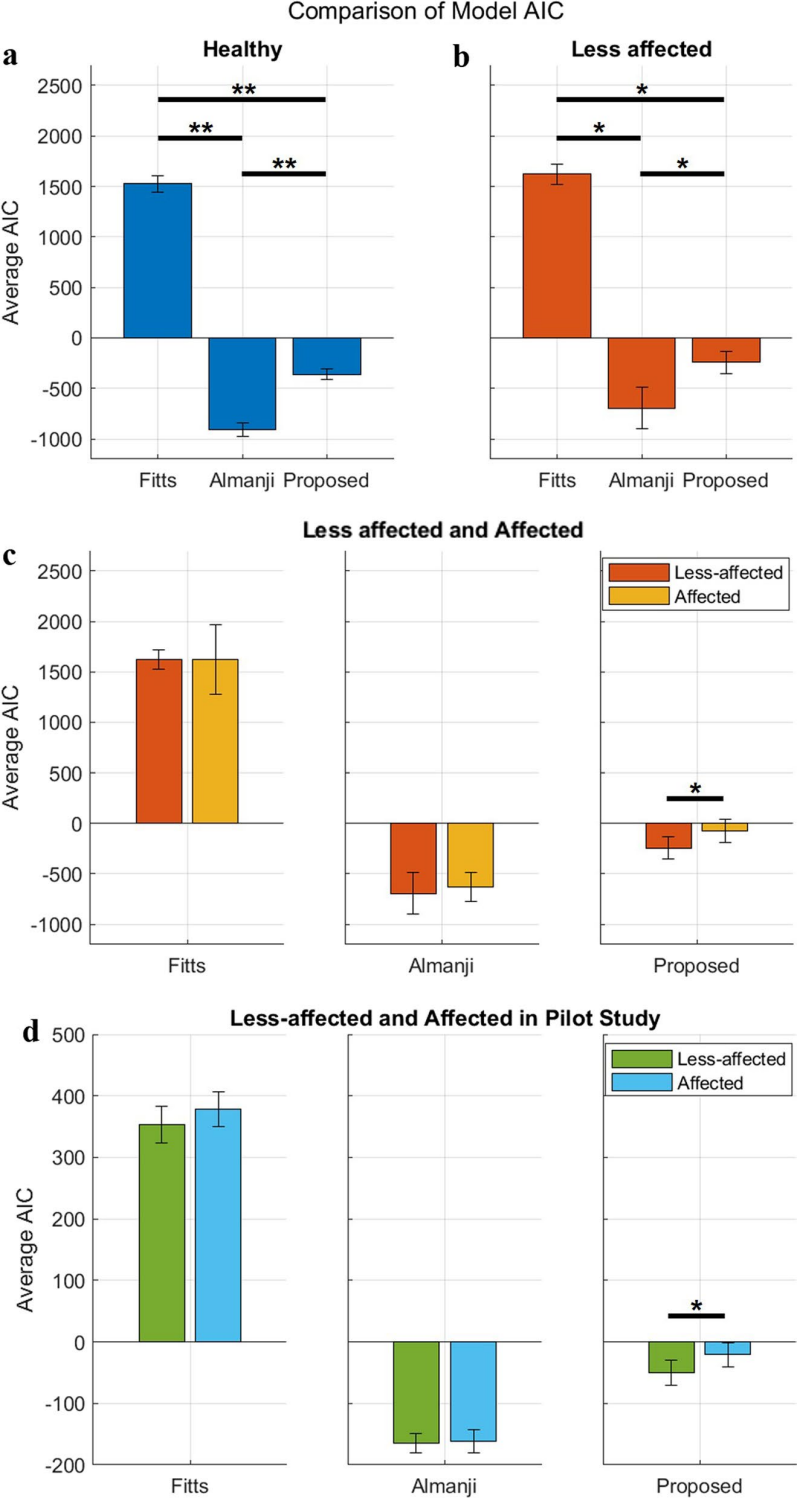
Comparison of average Akaike information criterion (AIC) for the candidate models. **a** and **b** show the model comparisons in the healthy and less-affected reaching movement (RM), respectively in the validation experiments. **c** Comparison of average AIC for the candidate models between the less-affected and affected arms within stroke patients in the validation experiments. **d** Comparison of average AIC for the candidate models between the less-affected and affected arms within the stroke patients in the pilot study (asterisks indicate significant differences; *p < 0.05, **p < 0.01)

Table 4 summarizes the AIC and *R*^*2*^ values for each candidate model in the validation experiments. As shown in Fig. 6a, the average AIC of all the models in the healthy reaching group was significantly different (*p* < 0.01). The proposed (4) and Almanji (3) models showed a negative AIC, whereas Fitts’ (1) model was the only model that resulted in a positive AIC (Fig. 6a). This trend between the models was also noted in the patients’ with less-affected reaching, for which all models were significantly different (*p* < 0.05) (Fig. 6b).

**Table 4:** AIC and R^2^ values of candidate models in validation experiments

Participants	AIC			*R*^2^		
	Fitts’	Almanji	Proposed	Fitts’	Almanji	Proposed
Healthy						
H1	1551	− 914	− 288	0.605	0.999	0.798
H2	1558	− 924	− 329	0.612	0.999	0.79
H3	1623	− 908	− 357	0.678	0.999	0.928
H4	1630	− 840	− 295	0.58	0.999	0.856
H5	1555	− 977	− 477	0.859	0.999	0.974
H6	1454	− 906	− 373	0.803	0.999	0.92
H7	1579	− 941	− 330	0.577	0.999	0.847
H8	1580	− 908	− 308	0.707	0.999	0.873
H9	1417	− 937	− 402	0.718	0.999	0.855
H10	1379	− 934	− 398	0.797	0.999	0.903
H11	1557	− 736	− 341	0.66	0.999	0.854
H12	1450	− 972	− 408	0.782	0.999	0.908
Stroke: Less-affected						
S1	1585	− 502	− 360	0.579	0.982	0.934
S2	1658	− 829	− 262	0.55	0.999	0.891
S3	1778	− 656	− 106	0.314	0.998	0.762
S4	1451	− 336	− 335	0.752	0.847	0.841
S5	1594	− 866	− 352	0.527	0.999	0.933
S6	1635	− 849	− 132	0.385	0.999	0.509
S7	1637	− 822	− 157	0.436	0.999	0.701
Stroke: Affected						
S1	1743	− 756	− 208	0.513	0.999	0.846
S2	1882	− 733	3	0.143	0.999	0.509
S3	1757	− 664	9	0.186	0.999	0.444
S4	1558	− 441	− 253	0.227	0.972	0.846
S5	893	− 425	− 50	0.005	0.999	0.45
S6	1885	− 648	54	0.111	0.999	0.339
S7	1645	− 748	− 80	0.39	0.999	0.565

AIC denotes Akaike information criterion; *R*^*2*^ denotes the coefficient of determination

Figure 6c compares the average AIC of each model in the less-affected and affected groups. Although Fitts and Almanji models showed no significant difference in the AIC between the less-affected and affected reaches, the proposed model showed a significant difference (*p* < 0.05; Fig. 6c). This implies that the predictive accuracy of the proposed model is affected by the presence of erroneous behavior, whereas Fitts and Almanji methods were not, which corresponds to the results of the pilot study (Table 5, Fig. 6d).

**Table 5:** AIC and R^2^ values of candidate models in pilot study

Participants	AIC			*R*^2^		
	Fitts’	Almanji	Proposed	Fitts’	Almanji	Proposed
Stroke: Less-affected						
P1	335	− 158	− 55	0.570	0.997	0.752
P2	349	− 176	− 72	0.860	0.999	0.959
P3	331	− 178	− 63	0.572	0.999	0.776
P4	350	− 188	− 71	0.774	0.999	0.941
P5	421	− 171	− 13	0.264	0.999	0.768
P6	395	− 170	− 26	0.321	0.999	0.783
P7	326	− 163	− 59	0.599	0.997	0.745
P8	363	− 130	− 20	0.479	0.996	0.515
P9	357	− 148	− 25	0.203	0.997	0.436
P10	367	− 178	− 54	0.385	0.999	0.872
P11	384	− 181	− 30	0.579	0.999	0.835
P12	358	− 170	− 57	0.584	0.999	0.875
Stroke: Affected						
P1	352	− 179	− 39	0.420	0.999	0.603
P2	419	− 121	8	0.398	0.998	0.505
P3	334	− 193	− 44	0.634	0.999	0.673
P4	391	− 177	− 30	0.570	0.999	0.828
P5	407	− 156	− 6	0.255	0.999	0.572
P6	353	− 166	− 48	0.584	0.999	0.814
P7	372	− 149	− 13	0.233	0.998	0.295
P8	384	− 150	− 12	0.160	0.999	0.446
P9	380	− 157	− 9	0.208	0.999	0.394
P10	362	− 174	− 38	0.480	0.999	0.769
P11	423	− 161	12	0.023	0.999	0.204
P12	362	− 149	− 31	0.635	0.999	0.733

AIC denotes Akaike information criterion; *R*^2^ denotes the coefficient of determination

#### Regression analysis

**Fig. 7:**
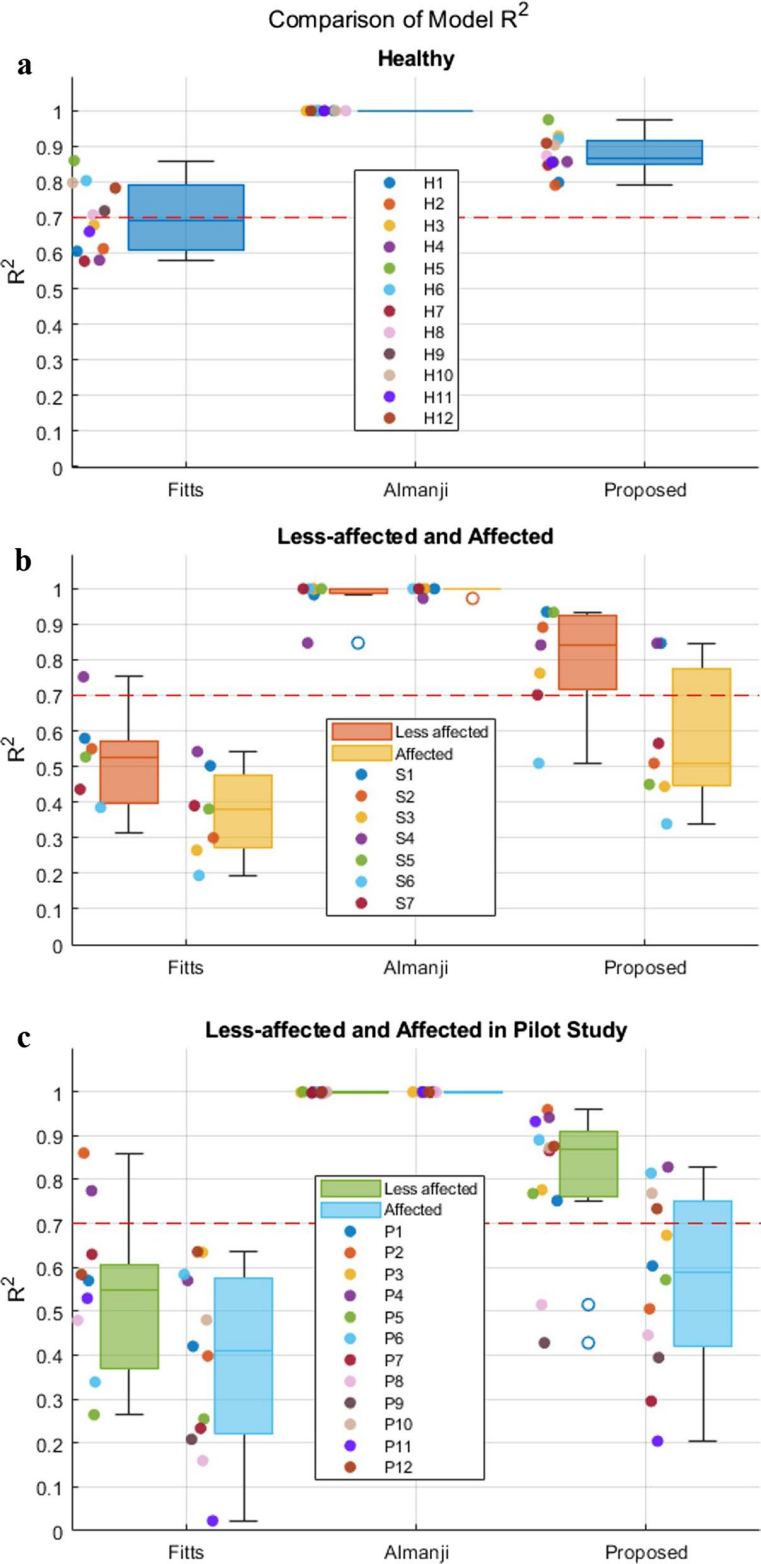
Comparison of model *R*^*2*^. The boxplot represents the distribution, and the scatter plot represents the actual *R*^*2*^. (**a**) and (**b**) present the *R*^*2*^ distributions in healthy models and between less-affected and affected models in the validation experiments, respectively. (**c**) The boxplot and scatter plot represent the actual *R*^*2*^ distribution in the pilot study. The red-dashed line represents *R*^2^ of 0.7 indicating a widely accepted level of strong model fitting (the higher the value, the better)

Regarding *R*^*2*^ in the healthy subjects in the validation experiments, both the Almanji and proposed models indicated a very strong fit for all subjects (*R*^*2*^ > 0.7) (Fig. 7a), whereas that of the Fitts’ model showed a strong fit for six out of 12 subjects (Fig. 7a). However, the *R*^*2*^ of the models for stroke differed from that of healthy reaching. For the less-affected RM, Fitts’ model had only one strong fit out seven subjects (Table 4, Fig. 7b). The Almanji (12 out of 12) and proposed (6 out of 7) models still resulted in an overall strong goodness of fit for the less-affected reaching data, which implies that both models are potential candidates for explaining the normal reaching of the less-affected arm. The overall trend was the same as that in the pilot study results. For the less-affected RM, Fitts’ model had only two strong fits out of 12 subjects (Table 5, Fig. 7c). The Almanji (12 out of 12) and proposed (10 out of 12) models still resulted in an overall strong goodness of fit for the less-affected RM data.

For the affected RM in the validation experiments, none of the Fitts’ models indicated a strong fit in terms of the *R*^*2*^ values, whereas the Almanji model resulted in a strong fit in all subjects (Table 4, Fig. 7b). The proposed models showed a strong fit in two out of the seven subjects. This suggests that the *R*^*2*^ of the proposed model was notably weakened in terms of fitting the erroneous reaching movement times. The Almanji model can also explain the erroneous behaviors, and it is undesirable for the intended normal reaching model. The pilot study result of the affected arm also indicated that the Almanji model resulted in a strong fit in all subjects, and the proposed model showed a strong fit in four out of 12 subjects (Table 5, Fig. 7c).

Table 6 summarizes the *R*^2^ values of the proposed model and average kinematic data for each post-stroke participant. The table presents the relationship between the severity of erroneous RM and the goodness of fit of the proposed model.

**Table 6:** *R*^2^ values of the proposed model and the average kinematic data for each post-stroke

Participants	Less-affected Arm					Affected Arm				
	*R*^2^	*T*_*M*_ [ms]	*AS* [m/s]	*CI*	*NS*	*R*^*2*^	*T*_*M*_	*AS* [m/s]	*CI*	*NS*
S1	0.934	1894	0.046	1.11	2.81	0.846	2233	0.042	1.16	3.11
S2	0.891	2221	0.040	1.12	2.61	0.509	2917	0.037	1.27	3.64
S3	0.762	2231	0.041	1.11	3.41	0.444	2332	0.046	1.33	3.74
S4	0.841	1669	0.057	1.1	2.77	0.846	1862	0.052	1.12	3.04
S5	0.933	1868	0.047	1.12	2.84	0.45	1755	0.060	1.47	3.63
S6	0.509	1755	0.055	1.28	3.34	0.339	2502	0.056	1.68	3.77
S7	0.701	1753	0.056	1.27	3.53	0.565	1867	0.056	1.37	3.78
P1	0.752	2766	0.041	1.14	3.29	0.603	2877	0.042	1.23	3.38
P2	0.959	3744	0.028	1.06	3.33	0.505	4978	0.033	1.75	3.48
P3	0.776	2689	0.044	1.21	2.83	0.673	2680	0.055	1.54	3.44
P4	0.941	3493	0.031	1.13	2.79	0.828	4628	0.024	1.14	3.31
P5	0.768	3682	0.037	1.34	3.46	0.572	3914	0.034	1.32	3.25
P6	0.783	3183	0.037	1.16	2.65	0.814	2896	0.043	1.27	3.15
P7	0.745	2517	0.044	1.14	2.83	0.295	2773	0.048	1.41	3.27
P8	0.515	2802	0.055	1.57	3.25	0.446	3066	0.059	1.92	3.06
P9	0.436	2215	0.061	1.41	3.09	0.394	2795	0.060	1.77	3.4
P10	0.872	2921	0.043	1.26	3.13	0.769	3136	0.040	1.28	3.13
P11	0.835	3786	0.034	1.26	3.27	0.204	3495	0.067	2.84	3.58
P12	0.875	3287	0.035	1.18	3.17	0.733	3209	0.042	1.39	3.38

*T*_*M*_ denotes movement time; *AS* the average speed; *CI* the curvature index; and *NS* denotes the number of submovements

#### Model fitting in erroneous RM (residuals)

**Fig. 8:**
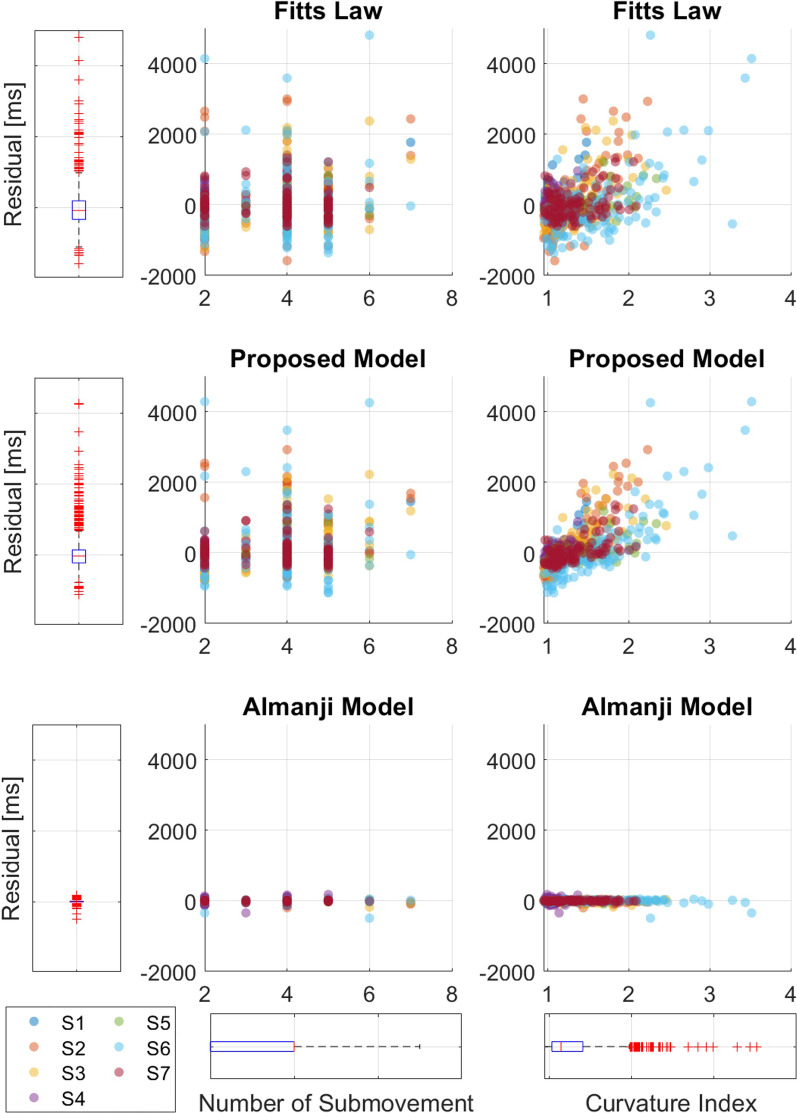
Relationship between residuals of each candidate model and erroneous reaching parameters from the affected side of post-stroke patients in the validation experiments. Erroneous reaching movement parameters are the number of submovment (*NS*) and curvature index (*CI*). Every reaching data point is projected to the boxplots to portray the distributions. In the presence of erroneous behaviors (high *NS* and *CI*), Fitts’ model and the proposed model formulated by the reaching data of the affected arms result in high model residuals, whereas the Almanji model have low model residuals

**Fig. 9:**
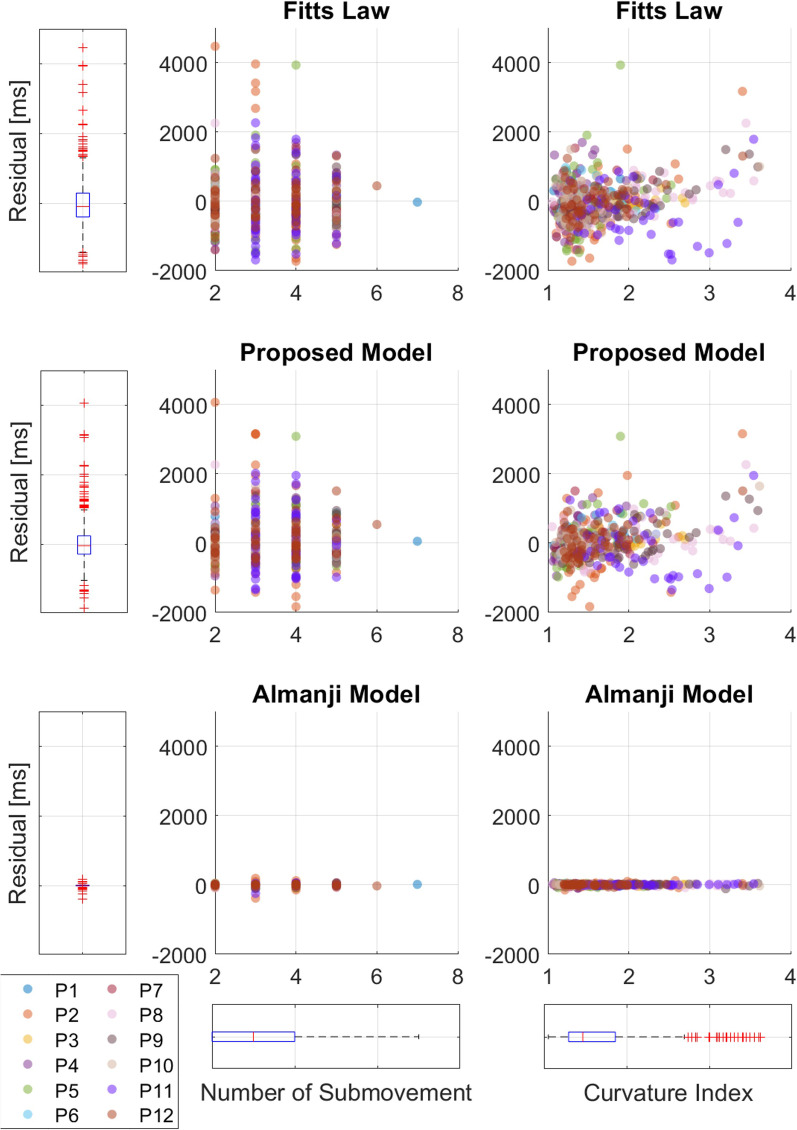
Relationship between residuals of each candidate model and erroneous reaching parameters from the affected side of post-stroke patients in the pilot study. Similar to the post-stroke data of the validation experiments, Fitts’ model and the proposed model formulated by the reaching data of the affected arms result in high model residuals, whereas the Almanji model have low model residuals

Considering the model performance for erroneous RM, Fig. 8 shows the relationship between the residuals of each model and the erroneous reaching-related parameters (*NS* and *CI*) in all the affected RM data. First, the bottommost boxplots presented in Fig. 8 show that *NS* and *CI* were mostly distributed over 2–7 and 1–3.5, respectively, suggesting that the affected RM data contained many erroneous reaching behaviors. The same data were projected onto boxplots of the model residuals on the left (Fig. 8). For Fitts’ model and the proposed model, the variances of the residuals were significant (Fig. 8), and the residual variances of all subjects were relatively large (650 and 590 ms, respectively). However, with negligible variance, the residual variance for Almanji was notably small (40 ms) compared to those of the other two models. From this, we interpret that the Almanji model cannot distinguish the affected reaching with erroneous behaviors from normal reaching, and we observed that the residual characteristics were the same in the pilot study (Fig. 9).

Overall, the proposed model is the most appropriate for the normal reaching model for the following reasons: (1) Fitts’ law was the least able to explain healthy and less-affected data among the candidate models; (2) the Almanji model best estimated the reaching movement time, followed by the proposed and Fitts’ models, but it even accurately captured the erroneous RM; (3) the proposed model had sufficient ability to explain the movement time for healthy and less-affected reaching, while the model did not explain the affected RM, as we intended.

### Evaluation visualization

**Fig. 10:**
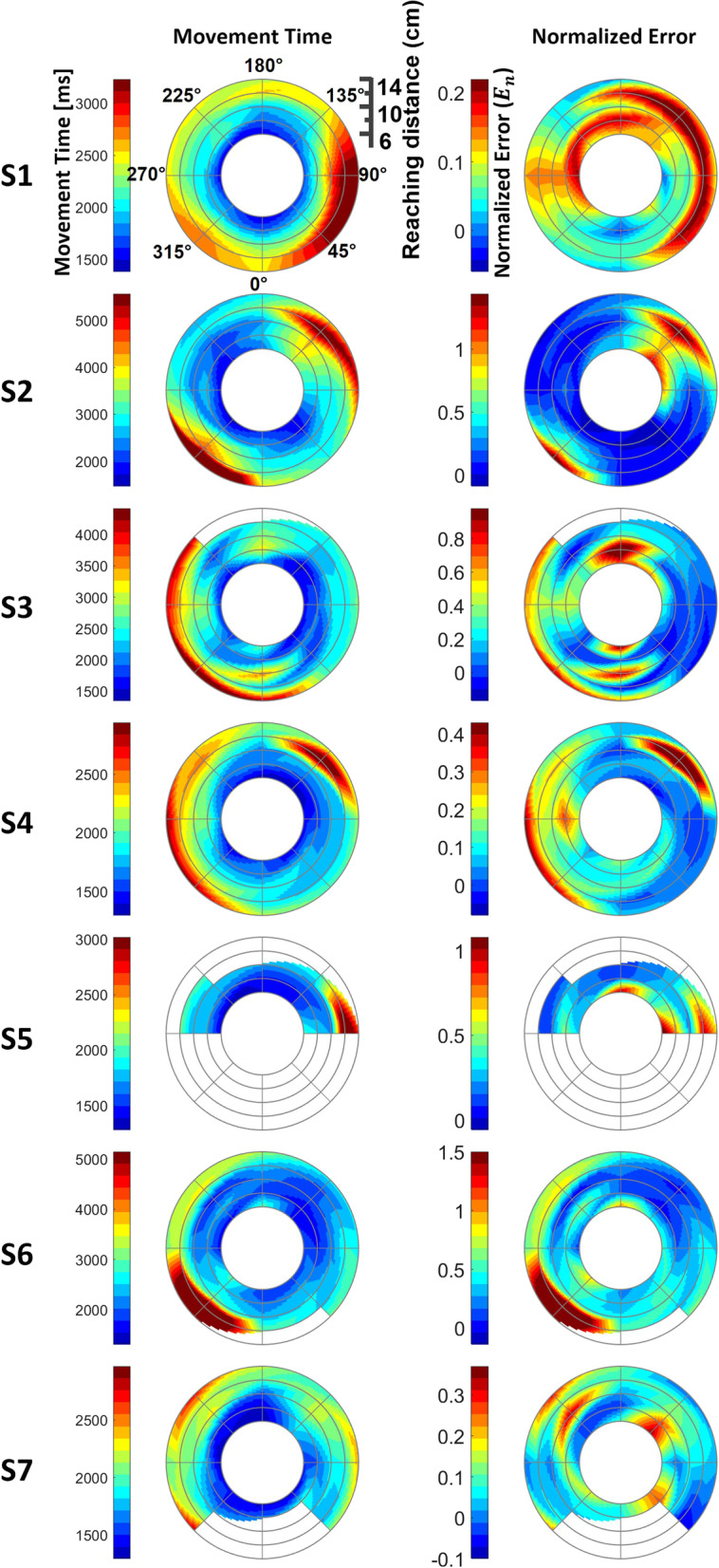
Reaching profile of post-stroke participants for the affected sides in the validation experiments. Each profile has the information of 40 targets distributed over five distances and eight directions grids in the reaching movement time and normalized error. Each subject had a different normalized error range. Non-colored areas represent unreachable targets

**Fig. 11:**
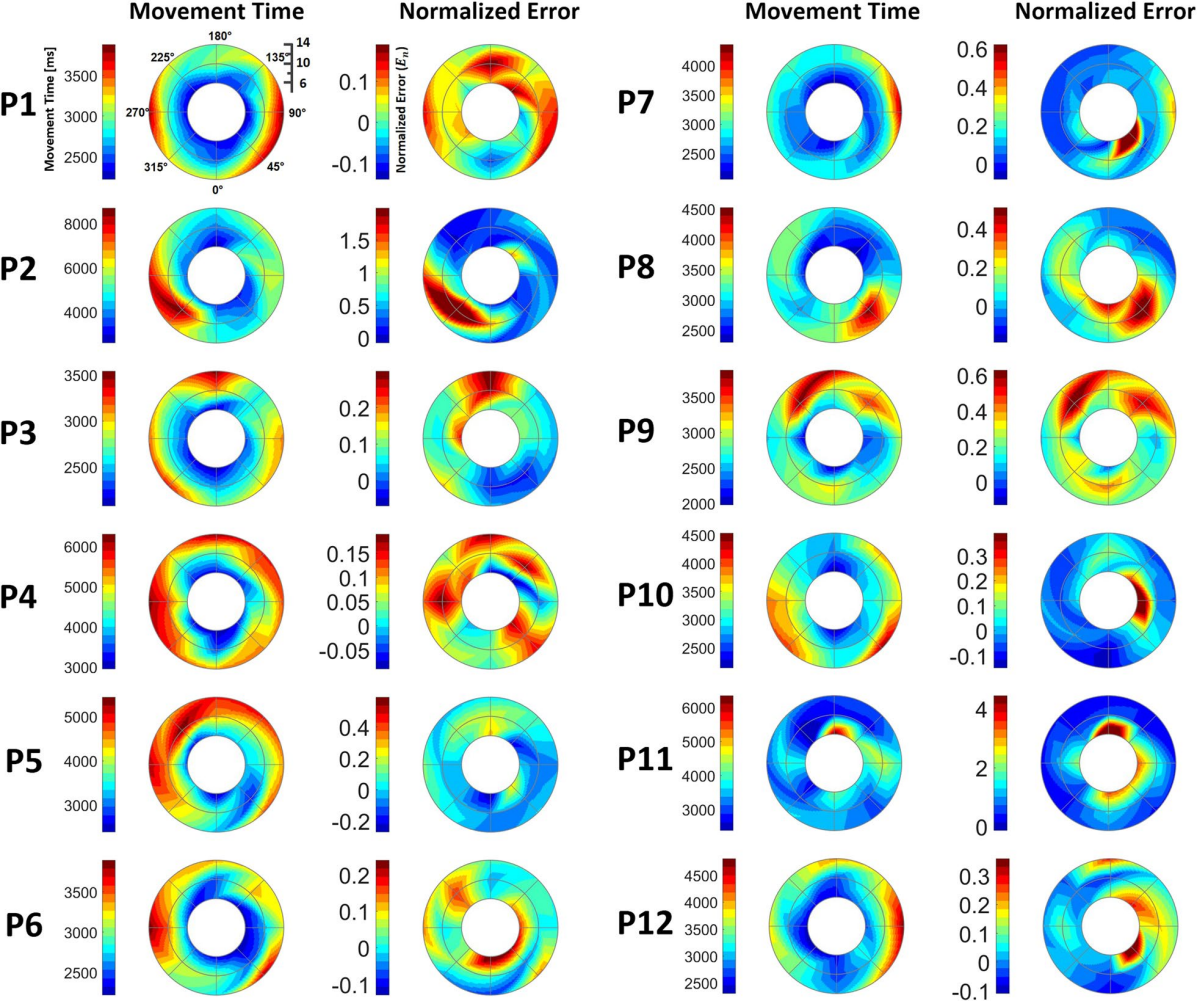
Reaching profile of post-stroke participants for affected sides in the pilot study. Each profile has the information of 24 targets distributed over three distances and eight directions grids in the reaching movement time and normalized error. Each subject had a different normalized error range

Based on the proposed model, we profiled the RM evaluation results of every post-stroke subject by contouring ${e}_{n}$ and ${T}_{m,a}$ for all movement conditions in the workspace (Figs. 10, 11). Each ${e}_{n}$ map of the affected side uniquely portrays the reaching characteristics of individuals at specific distances and directions, in which erroneous reaching was emphasized in yellow and red (Figs. 10 and 11). The movement time maps using ${T}_{m,a}$ portrayed a tendency of higher ${T}_{m,a}$ on the targets of further distances, and the corresponding ${e}_{n}$ map still captured the affected movements in the targets with shorter distances (S1, S2, S3, S4, and S7 in Fig. 10; all participants except P11 in Fig. 11). Notably, all individuals had different scales of the spectrum range for the contours, which reflected the inter-subject severity of the affected arm. Specifically, S1 and S7 in Fig. 10 and P1, P4 and P6 in Fig. 11 have comparably small scales; therefore, the affected maps seem to have abnormal motor characteristics globally. S5 shows a case in which the affected RM data were severely impaired with a significantly constrained workspace (Fig. 10). The visualization clearly shows that he could not reach more than half of the targets, particularly those near the trunk.

## Discussion

In this study, we developed a novel, individually scaled RM evaluation method. We first determined that the proposed reaching model (4) is the most appropriate for identifying normal reaching and is valid for describing both healthy and less-affected RM. Using (4) and (5), we intuitively characterized the impaired (affected) reaching condition through the discrepancy between the actual RM performance and the individual’s predicted normal performance.

Several statistical goal-directed movement models are applicable to describing RM. However, they are not fully compatible for distilling an individual’s normal reaching model [24, 26]. One study utilized Fitts’ law to assess an individual’s reaching ability to control reaching training difficulty, but its reliability was inconsistent among the participants for the less-affected and affected arms [27]. This result corresponded to our results (Fig. 7b, and c); Fitts’ law was unsuitable for describing the normal and less-affected RM than the other models. It is noteworthy that the validity of Fitts’ law in a multi-directional environment is still contradictory depending on the studies [44, 45], however, Fitts’ law is representative of goal-directed movements as a candidate model [24, 46]. Another study reported a reaching-related (Almanji) model that consisted of sophisticated human effect variables related to mouse click movement in CP, not stroke reaching [26]. The Almanji model could be modified to (3) to explain the RM, and we observed that this model had the strongest prediction accuracy for explaining individual reaching (Figs. 6 and 7). However, the Almanji model could accurately fit the affected RM, which opposes the purpose of the intended normal reaching model (Fig. 7b and c). Further analysis indicated that the Almanji model predicted the movement time of the affected RM too accurately regardless of *NS* and *CI*, which are related to erroneous reaching (Figs. 8 and 9).

In this study, we deduced that normal reaching can be explained using the proposed model, which consists of *AS* and the target distance only (Figs. 6a and 7a). This finding could also be supported by the Almanji study for point-click tasks; the model components for healthy subjects were governed by the *AS*, *CI,* and target distance [26]: therefore, ideally performed RM could be modeled without *CI*.

**Fig. 12:**
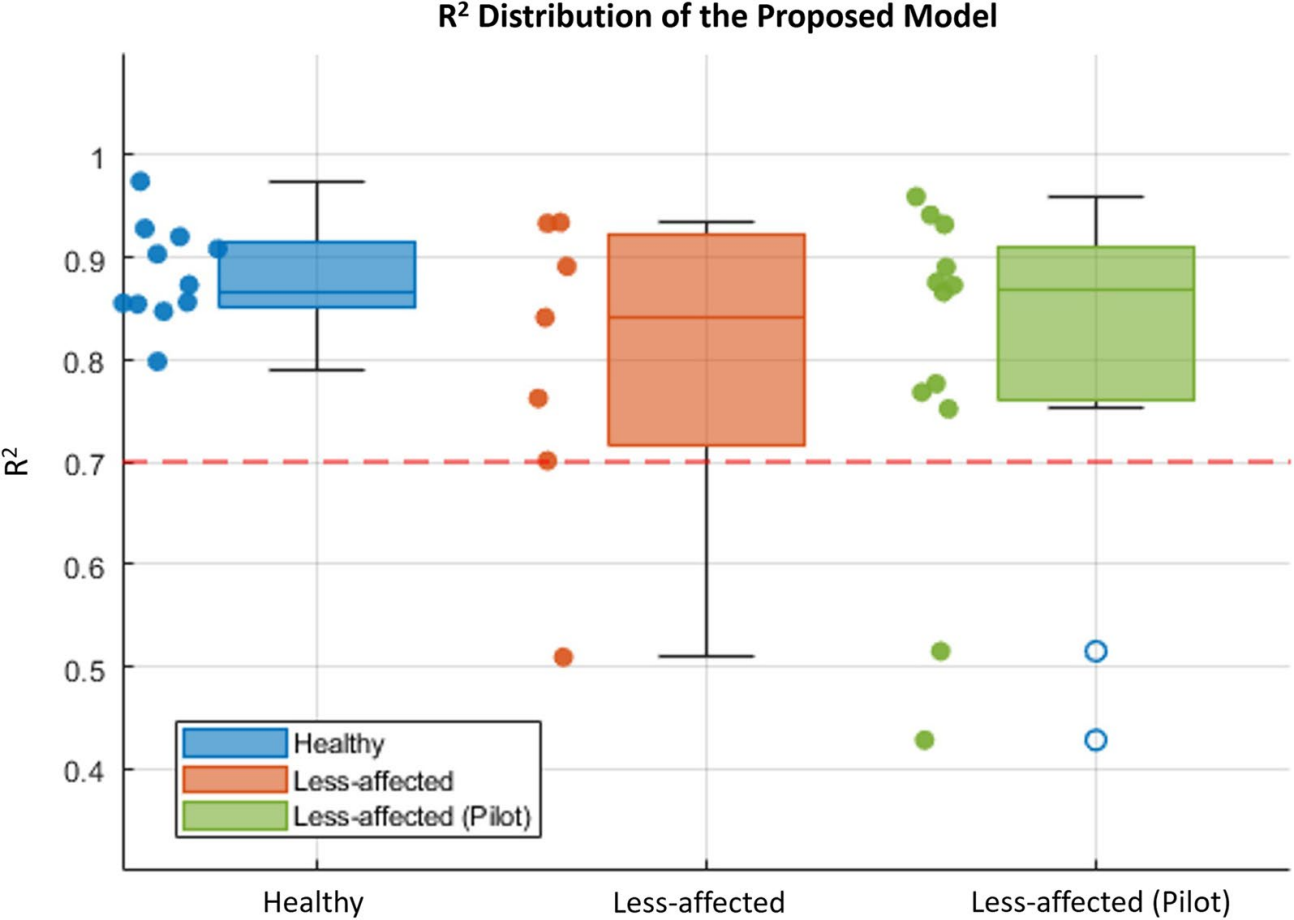
*R*^*2*^ distribution of the proposed model in healthy and less-affected conditions (redrawn from Fig. 7)

When the normalized error ${e}_{n}$ of (5) was developed, we assumed that the less-affected reaching could be regarded as a reliable source of the individuals’ normal reaching ability. Figure 12 summarizes the *R*^*2*^ results of the proposed model for the normal (healthy) and less-affected reaching in both the validation experiments and pilot study. The proposed model obtained from less-affected reaching was sufficient for normal reaching. Most *R*^*2*^ values (16 of 19) lie near 0.7, which shows a strong fit of the regression model in both the validation experiment and pilot study (Fig. 12). Even the weak cases of the model (S6: *R*^*2*^ = 0.509, *p* < 0.001; P8: *R*^*2*^ = 0.515, *p* < 0.001; P9: *R*^*2*^ = 0.436, *p* < 0.001) also showed a moderately good fit [47] (Table 6, Fig. 12). It should be noted that the weakest fit in the less-affected reaching (S6) of the validation experiment would come from a large variation in *CI* (1.23 ± 0.25) and *NS* (3.48 ± $1$.22) for which, in fact, both variances were the largest among all less-affected reaching for stroke participants in the validation experiment (Table 6). Similarly, P8 and P9 in the pilot study showed large variations in *CI* (P8: 1.57 ± 0.45, P9: 1.41 ± 0.28) and *NS* (P8: 3.25 ± 1.07, P9: 3.09 ± 0.90). These weak-case scenarios show the potential limitations of the proposed method when both arms are notably affected. It is noteworthy that the reaching performance between the less-affected arm of the post-stroke and the normal movement of the typical control group could differ depending on the severity of the patients. Some studies reported that the less-affected arm has deficits in cases of more severe and acute participants [48–50], whereas the mild to moderate stroke group showed no discrepancies in kinematics compared to the healthy control group [21–23].

After establishing the normal reaching model, we used the normalized error (5) as the index to evaluate the affected RM for the following reasons: (1) The index intuitively presents the discrepancy between the actual reaching performance and model-predicted normal performance, which can reflect the degree of abnormality. Moreover, (2) it is a consolidated single parameter that consists of multidimensional information, such as the target distance, *AS*, *CI,* and *NS,* making it easier to interpret. In addition, (3) thanks to the normalization, the index, which is no longer biased to the effect of the target distance, is especially advantageous in a multi-distance-directional reaching training environment.

Several studies have reported evaluation methods for spatial profiling the reaching characteristics using indices (i.e. movement time); these visual representations have been used to prescribe adaptive training according to individual characteristics and evaluate performance changes before and after reaching training [4, 9, 19, 20]. In this study, we extended the performance mapping methods using a model-based index (5), ${e}_{n}$. Mapping only the RM performance with movement time can be problematic in a multi-distance-directional environment because a short movement time is generally preferred. Because further targets are likely to have long movement times, the training prescription based on the movement time could be biased toward the outer-most targets regardless of the actual RM performance in the workspace (Figs. 10 and 11). In contrast, the proposed method based on the normalized error ${e}_{n}$ can objectively portray the RM performance globally in a spatial sense regardless of the distance (Figs. 10 and 11), allowing us to objectively prescribe training priorities in the reaching environment.

Here, we presented only static characteristics by mapping, but the method could be extended to address temporal changes in the characteristics as well. For instance, during RM training, changes in index ${e}_{n}$ (5) can be observed, as a previous study demonstrated a learning/fatigue model in the movement time within a reaching training session [51]. However, the sole movement time does not provide a cue on when to terminate the training, whereas the normalized error provides a sense of desired recovery. Therefore, if a subset that reaches the target achieves the desired recovery (small ${e}_{n}$), prospective training can prescribe different subsets that maximize the recovery opportunities.

Our evaluation method was primarily developed by considering robot-aided RM training; however, this application could be extended to general reaching training because it utilizes only the kinematic data of RM. In addition to robots, sensors that can measure and estimate the kinematic data of the hand, such as the touch screen, red/green/blue and depth sensors, and inertial measuring units, are feasible for implementing the evaluation method [8, 51–55]. Hence, the proposed evaluation method can improve conventional reaching training using quantifiable records obtained from sensors. As an eventual goal, the proposed evaluation method can enable individualized robot-aided RM training.

This study has several limitations. First, the number of trials required to construct a normal RM model was not optimized. In the validation experiments, 120 reaching trials were used for concrete validation, and we conducted 24 and 48 trials for the pilot study. In future, we need to develop a systematic method for determining the number to form a valid normal RM for clinical applications. Next, the normal reaching model could be unsuitable for evaluating the affected RM if the less-affected arm of hemiplegic patients showed less model reliability as shown in S6 (Table 4). Although this was an uncommon case, we may need to develop additional algorithms (i.e., clustering) to detect normal reaching trials more selectively and enhance the feasibility of the proposed method. Finally, our results were verified using a limited number of participants (12 healthy and 19 stroke patients). Hence, further studies with larger populations could enhance the validity of the proposed method against unconsidered kinematic variabilities, such as the effect of arm dominance, with sufficient statistical power.

## Conclusions

In this study, we developed a novel individually scaled RM evaluation method based on a normal reaching model. We first validated that the proposed reaching model can determine the normal RM of both healthy and less-affected RM. Using the proposed index, we could intuitively visualize the individual (affected) RM condition through the discrepancy between the actual RM performance and individual’s predicted normal performance. Furthermore, our method has the potential to provide effective adaptive training by prioritizing a set of reaching movements spatially and terminating satisfactory reaching movements.

## Data Availability

Data and materials can be made available upon request to the authors.

## Abbreviations

AIC: Akaike information criterion
AS: Average speed
CI: Curvature index
CP: Cerebral palsy
EC: Erroneous clicks
FMA-UE: Fugl-Meyer Upper Extremity Assessment
H: Hemorrhagic
I: Ischemic
MAS: Modified Ashworth scale
MBC: The modified Brunnstrom classification
MMT: Manual muscle test
NS: Number of submovements
NSO: Number of slip-offs
RM: Reaching movement
